# A kinetic and thermodynamic investigation into the removal of methyl orange from wastewater utilizing fly ash in different process configurations

**DOI:** 10.1007/s10653-020-00567-6

**Published:** 2020-05-11

**Authors:** J. H. Potgieter, C. Pardesi, S. Pearson

**Affiliations:** 1 School of Chemical and Metallurgical Engineering, The University of the Witwatersrand, 1 Jan Smuts Avenue & Jorrissen Street, Johannesburg, 2050 South Africa; 2 Manchester Metropolitan University, Manchester, UK

**Keywords:** Methyl orange, Coal fly ash, Adsorption studies, Batch, Column, Heap adsorption

## Abstract

The removal of methyl orange using coal fly ash, which is a widely available low-cost adsorbent, has been investigated. Adsorption studies for dye removal were conducted using various configurations such as batch, column and heap adsorption at various temperatures and adsorbent dosages at neutral pH. The Langmuir, Freundlich and Tempkin isotherm models were used to describe the process. The Freundlich model best represented the adsorption. Kinetic studies show the adsorption followed pseudo-second-order kinetics. Thermodynamic studies show that the process is spontaneous, endothermic and random. Column configuration was found to be the most efficient with a dye removal percentage of 99.95%, followed by heap adsorption at 99.25% removal and lastly batch configuration with 96.68% removal. Economic analysis shows that column operation would be the most effective for practical implementation.

## Introduction

A very important aesthetic quality of textiles in the modern world today is colour. Manufacturing and the use of various dyes are well-established industrial processes. However, the toxic nature of these dyes is becoming a growing environmental concern which needs to be addressed. Synthetic dyes are released from a variety of industries such as textiles, paper, pulp and dyestuff manufacturing (Banerjee et al. 2014), making them one of the largest environmental polluters. Sun et al. (2010) reports that dyes account for approximately 100 tons of waste annually. According to the World Health Organization, by 2025 half of the world’s population will be living in water-scare areas (WHO 2017). Given these current water shortage problems and poor water quality, especially in Africa, the organic pollutants released from residual dye effluents are threatening the long-term sustainability of water. In addition to dye effluents being aesthetically unappealing, they have high chemical and biological oxygen demands (Robinson et al. 2001). These organic pollutants are toxic to aquatic life as potentially carcinogenic and mutagenic compounds are produced which are exceptionally harmful to the environment (Banat et al. 1996). Given these harmful effects, dye removal techniques are an essential and necessary step in wastewater treatment.

Residual dyes in wastewater effluents make conventional treatment methods difficult for numerous reasons, owing to their complex aromatic structure which is resistant to microbial degradation. This can result in treated effluents still being highly coloured (Visa et al. 2010). Multiple treatment methods are commercially available, and one such method that is commonly implemented is adsorption. It has been proved to be one of the most effective ways of colour removal due to simplicity and ease of operation (Ghaedi et al. 2012). However, adsorbents such as activated carbon are often costly, making this technique unattractive. This has promoted research into the use of other low-cost adsorbents such as coal fly ash. Coal fly ash, which is a by-product produced during the combustion of coal in the electricity generation industry accounts for million tons of waste annually worldwide, and in South Africa alone amounts to more than 30 million tons annually (Escom 2017). If viable for colour removal, it would be an excellent low-cost adsorbent. This adsorbent is available in abundance and is creating an environmental concern by being disposed of in landfill sites. Coal fly ash is currently being investigated as an adsorbent using batch configuration under various operating conditions such a pH, temperature, stirring speed, adsorbent dosage and initial dye concentration. It was found to have good adsorption capacity due to the presence of SiO_2_, TiO_2_ and the remaining unburnt carbon content in the ash (Mohan et al. 2002; Wang and Wu 2006). Studies have shown that pre-treatment of the coal fly ash using physical and chemical methods can improve the adsorption capacity, although this would increase the operating costs of such procedures (Wang and Zhu 2005). Banerjee et al. (2014) investigated the adsorption of methylene blue using acid-activated coal fly ash, which was found to have a high adsorption capacity. Wang et al. (2006) evaluated the effect of NaOH to modify coal fly ash for methylene blue removal and found a 25% increase in its adsorption capacity.

Dye removal techniques have been researched extensively over the past few years; however, very few of these techniques have been implemented by wastewater treatment companies owing to the high cost, low efficiency and inapplicability for a variety of dyes. The current research aims to address this problem and pose a cost-effective, efficient manner of colour removal using a cheap, widely available adsorbent.

This work is beneficial as the removal of an anionic dye has seldom been investigated. Batch configurations have been investigated extensively; however, few studies have been conducted to investigate the adsorption capacity using a continuous column operation or heap adsorption. These operating configurations could potentially have lower operating costs, higher adsorption capacities and be more efficient on a larger scale. The present work also determined the process activation energy, thermodynamic parameters, adsorption isotherms and kinetics using methyl orange (MO) dye.

## Materials and methods

### Adsorbate (methyl orange)

All chemicals and reagents used were of analytical reagent (AR) grade and were used without further purification. MO is an anionic azo dye available as bright orange crystals and was obtained from ACE Chemicals. MO stock solution (1000 mg l^−1^) was prepared by dissolving 1 g in 1000 ml of distilled water, and working solutions of 1–14 mg l^−1^ were prepared daily using serial dilution.

### Adsorbent (coal fly ash)

Coal fly ash was obtained from the Lethabo power station in South Africa. The coal fly ash was washed with distilled water to remove soluble inorganic material and surface dust particles. Characterization of the coal fly ash was done through XRD and XRF. The elemental composition is summarized in Table 1.

**Table 1:** Lethabo fly ash composition

Composition	Percentage (%)
SiO_2_	52.59
Al_2_O_3_	34.59
Fe_2_O_3_	3.15
TiO_2_	1.68
MnO	0.04
MgO	1.06
CaO	4.08
Na_2_O	0.17
K_2_O	0.60
P_2_O_5_	0.28
Cr_2_O_3_	0.04
NiO	0.02
V_2_O_5_	0.04
ZrO_2_	0.08
LOI	1.4

**Fig. 1:**
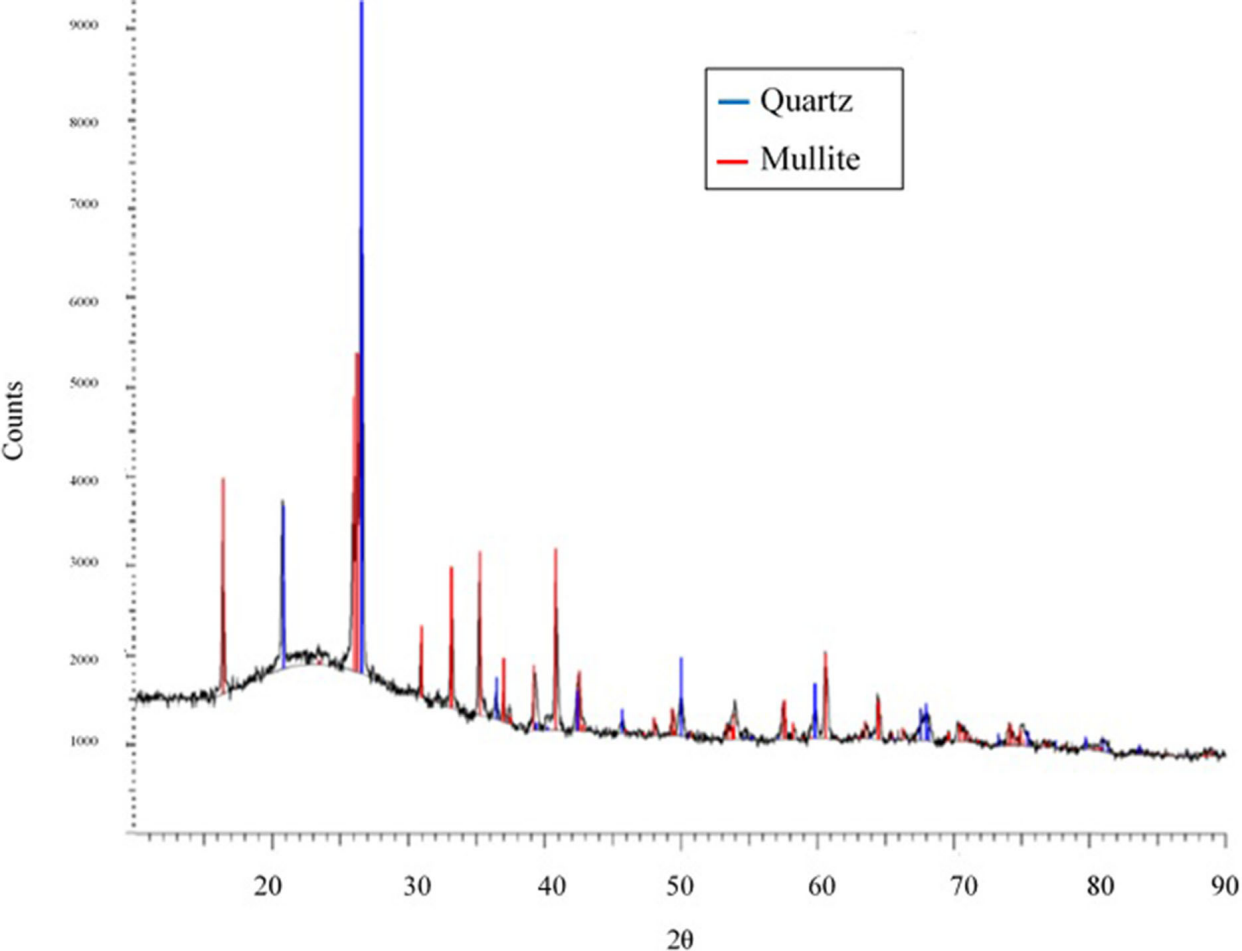
XRD Analysis of Lethabo fly ash

XRD analysis was conducted using a Bruker D2 Phaser X-ray Diffractometer quipped with Cu K $\alpha$ X-Ray source operating at 30 kV and 10 mA. From Fig. 1, the XRD pattern indicates that the mineral phases of the coal fly ash consist of quartz and mullite. The broad peak observed between 2 $\theta$ = 2° and 2 $\theta$ = 30° indicates that the amorphous phases in the coal fly ash contributes more than 50% of the total mass. This amorphous phases consists of silica and alumina in a glass matrix. The Lethabo coal fly ash has been analysed with regards to only major and minor elements. The analysis has not considered the effect of trace amounts of heavy metals. However, the coal fly ash does not contain any significant amounts of heavy metals or radioactive substances. The presence of these elements is dependent on the geological source of the coal used. Specifically, in South Africa this coal fly ash is widely used in the cement making industry, thus implying it is safe to use (Potgieter et al. 2002). Studies have also found that during the cement making process, cement addition had positive effect on the immobilization of heavy metals. However, heavy metal leaching is not a concern in the treated wastewater, except if the pH of the effluent is highly acidic (Wang et al. 2016; Madzivire et al. 2017; Izquierdo and Querol 2012).

Analysis of Lethabo coal fly ash was done by Van der Merwe et al. (2014). Particle size distribution analysis found the mean particle size to be 4.9 μm with 90% of the sample by volume having a particle size smaller than 14.9 μm. The surface area found using Brunauer–Emmett–Teller (BET) analysis was found to be 1.52 m^2 g−1^.

## 0.2.3 Batch adsorption tests

Batch adsorption tests were conducted using 5 g of coal fly ash in 150 ml of dye solution at a solid-to-liquid ratio of 1:30. The mixture was continually agitated at a speed of 400 rpm using a magnetic stirrer. Continuous readings were taken for an hour and equilibrium readings after a period of 24 h. The mixture was centrifuged to remove residual adsorbent particles and the absorbance measured. Residual dye concentrations were calculated using a calibration curve prepared at the maximum wavelength of 464 nm with a SQ-2800 UV–Vis spectrophotometer.

Dye concentrations below 4.3 × 10^–2^ mM were prepared to ensure that the absorbance remained below 1 and within the linear range of the calibration curve. This process was repeated over a range of temperatures (25–45 °C) in order to determine the rate constants and order of the reaction. Adsorption isotherms were determined in a similar manner by varying the absorbent dosage from 3–20 g. All other parameters such as pH, temperature and agitation speed remained constant, and only one variable was varied at a time. The amount of dye adsorbed at equilibrium (*q*_e_) as well as the removal percentages were calculated using Eqs. (1) and (2)

1 $$q_{{\text{e}}} = \frac{{\left( {C_{{\text{o}}} - C_{{\text{e}}} } \right)V_{{{\text{Sol}}}} }}{W}$$

2 $${\text{Removal}}\; \% = \frac{{\left( {C_{{\text{o}}} - C_{{\text{e}}} } \right)}}{{C_{{\text{O}}} }} \times 100\%$$

where *C*_o_ and *C*_e_ are the initial and equilibrium dye concentrations (mg l^−1^), *W* is the mass of adsorbent (g) and *V* is the volume of dye solution (l). Similarly, from the batch adsorption tests the adsorption kinetics, isotherms and thermodynamic parameters can be determined.

### Column adsorption tests

Column adsorption tests were conducted using the same dye concentrations as that of the batch tests. Columns were set up using burettes packed with coal fly ash, ranging from 3–20 g, of varying bed heights between 5 and 40 cm. The column had an internal diameter 1.5 cm. Dye solution was continuously added and allowed to pass through the column. A solid-to-liquid ratio of 1:5 was used.

### ***Hea***p adsorption tests

Heap adsorption tests were conducted using 180 g of coal fly ash. Dye solution was continuously sprayed on the surface of the coal fly ash. Three washing cycles were used per batch of coal fly ash with a solid-to-liquid ratio of 1:5.

## Results and discussion

Preliminary experimental runs were conducted in triplicate to ensure reproducibility and due to good reproducibility; the remaining experiments were conducted in duplicate and mean data values were used for analysis. A preliminary experimental run was also conducted under UV light; however, no significant change in results was observed. The latter experimental runs were conducted at ambient conditions.

### Effect of temperature

**Fig. 2:**
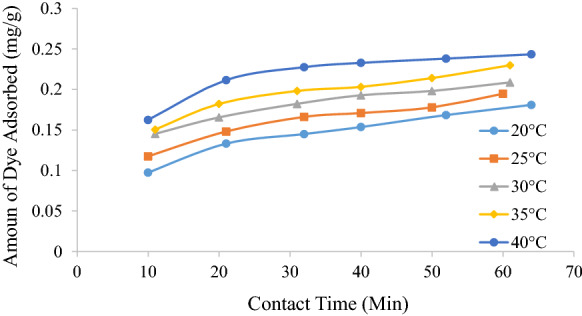
Effect of temperature of MO adsorption on fly ash

Batch adsorption tests were conducted in the range of 20–40 °C. Figure 2 shows that the dye removal percentages increase with an increase in temperature which indicates that the adsorption process is endothermic in nature. This may be attributed to the increased mobility of the dye molecules and an increase in the number of active sites due to the temperature increase. Higher temperatures are also known to cause an enlargement of pore size of the remaining active carbon present in the coal fly ash, which increases the adsorption capacity (Senthilkumaar et al. 2006).

### Adsorption kinetics

**Fig. 3:**
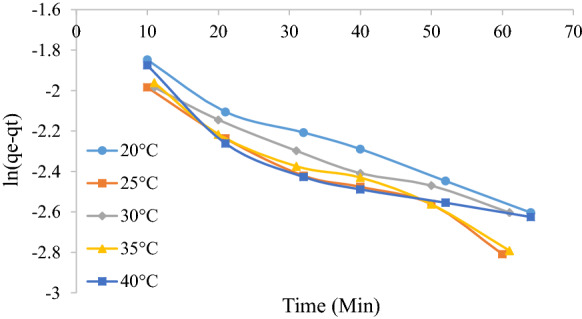
Lagergren first-order plot for the adsorption of MO at various temperatures

**Fig. 4:**
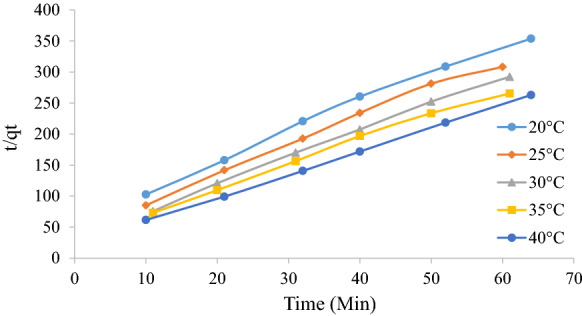
Lagergren pseudo-second-order plot for the adsorption of MO at various temperatures

Batch adsorption tests were used to determine the adsorption kinetics, namely whether the process follows Lagergren pseudo-first-order kinetics (Lagergren 1898) or pseudo-second-order kinetics (Ho and McKay 1999) as evaluated according to Eqs. (3) and (4), respectively.

3 $$\ln \left( {q_{{\text{e}}} - q_{{\text{t}}} } \right) = \ln \left( {q_{{\text{e}}} } \right) - k_{1} t$$

4 $$\frac{t}{{q_{{\text{t}}} }} = \left( {\frac{1}{{k_{2} }}} \right)\left( {\frac{1}{{q_{{\text{e}}}^{2} }}} \right) + \frac{1}{{q_{{\text{e}}} }}t$$

where *q*_e_ is the amount of dye adsorbed at equilibrium (mg g^−1^), *q*_t_ is the amount of dye adsorbed at time t (mg g^−1^), *k*_1_ is the pseudo first order rate constant (min^−1^), and *k*_2_ is second order rate constant (g mg^−1^ min^−1^). A linear plot of $\ln \left( {q_{{\text{e}}} - q_{{\text{t}}} } \right)$ versus $t$ will yield a straight line of slope equal to $k_{1}$ and intercept equal to $\ln \left( {q_{{\text{e}}} } \right)$ if the adsorption follows pseudo-first-order kinetics. A linear plot of $\frac{t}{{q_{t} }}$ versus $t$ will yield a straight line of slope equal to $\frac{1}{{q_{{\text{e}}} }}$ and intercept equal to $\frac{1}{{k_{2} q_{{\text{e}}}^{2} }}$ if the adsorption follows pseudo-second-order kinetics. Lagergren plots for different temperatures were plotted in Figs. 3 and 4 below. It can be seen that the pseudo-second-order plot follows a linear profile the entire operating period with a good correlation coefficient (*R*^2^ > 0.99). The first-order plot does not follow a linear profile. This is expected as generally only the initial contact period of approximately 30 min follows a linear relationship (Umpuch and Sakaew 2013). It is also seen that the rate constant decreases with an increase in temperature which is expected for physisorption processes (Tunali et al. 2006). Table 2 summarizes the Lagergren model parameters.

**Table 2:** Lagergren first-order and pseudo-second-order kinetic adsorption parameters

	Lagergren first order			Lagergren pseudo second order		
	*k*_1_ (min^−1^)	*R*^2^	*q*_e_	*k*_2_ (g mg^−1^ min^−1^)	*R*^2^	*q*_e_
20 °C	0.013	0.9805	0.17	0.33	0.9929	0.22
25 °C	0.017	0.9671	0.15	0.27	0.9909	0.24
30 °C	0.012	0.9796	0.15	0.23	0.998	0.26
35 °C	0.015	0.9674	0.16	0.21	0.9951	0.27
40 °C	0.12	0.8379	0.15	0.18	0.9996	0.30

### Adsorption isotherms

Adsorption studies are important for solid–liquid processes as this determines the interaction between the adsorbent and adsorbate. The adsorption isotherms were analysed according to the Langmuir, Freundlich and Tempkin models.

#### Langmuir isotherm

The Langmuir model is used to model monolayer and uniform adsorption on finite adsorption sites. It is also assumed that the adsorbed molecules do not interact with neighbouring sites ( Belhachemi and Addoun 2011). The Langmuir model is represented by Eq. (5) (Langmuir 1916).

5 $$\frac{{C_{{\text{e}}} }}{{q_{{\text{e}}} }} = \frac{{C_{{\text{e}}} }}{{q_{{\text{m}}} }} + \frac{1}{{k_{{\text{L}}} q_{{\text{m}}} }}$$

where *q*_e_ is the maximum adsorption capacity at equilibrium (mg g^−1^), *C*_e_ is equilibrium concentrations of the adsorbate (mg l^−1^), *q*_m_ is the theoretical maximum adsorption capacity for the process (mg g^−1^), *k*_L_ is the Langmuir adsorption constant related to the free energy of adsorption (l mg^−1^). A plot of $\frac{{C_{{\text{e}}} }}{{q_{{\text{e}}} }}$ versus $C_{e}$ will yield a straight line of slope equal to $\frac{1}{{q_{{\text{m}}} }}$ and intercept equal to $\frac{1}{{k_{{\text{L}}} q_{{\text{m}}} }}$ if Langmuir adsorption prevails.

#### *Freundlich* isotherm

The Freundlich model thoroughly represents multilayer adsorption on heterogeneous surfaces. It is not restricted to the formation of the monolayer and is represented by Eq. (6) (Freundlich 1906).

6 $$\ln q_{{\text{e}}} = \ln K_{{\text{F}}} + \left( \frac{1}{n} \right)\ln C_{{\text{e}}}$$

where *K*_F_ is a Freundlich constant indicative of the relative adsorption capacity of the adsorbent (mg g^−1^), $\frac{1}{n}$ is the heterogeneity factor indicating the favourability and capacity of the system (Piccin et al. 2011). A value of *n* < 1 indicates unfavourable adsorption, *n* > 1 indicates favourable adsorption and equal to 1 indicates the adsorption process is linear. A plot of $\ln q_{{\text{e}}}$ versus $\ln C_{{\text{e}}}$ will yield a straight line of slope equal to $\frac{1}{n}$ and intercept equal to ${\ln}K_{{\text{F}}}$ from which the constants can be determined if the process obeys the Freundlich isotherm.

#### *Temkin* isotherm

The Temkin model considers the effects of some indirect adsorbate/adsorbate interactions in adsorption isotherms (Piccin et al. 2011). The Temkin model is presented by Eq. (7).

7 $$q_{{\text{e}}} = \frac{RT}{b}\ln K_{{\text{T}}} + \frac{RT}{b}\ln C_{{\text{e}}}$$

where KT is the equilibrium binding constant (l mol^−1^), $b$ is a constant related to adsorption heat (J mol^−1^), *R* is the universal gas constant (8.314 J mol^−1 K−1^), and *T* is the absolute temperature (K). A plot of $q_{{\text{e}}}$ versus $\ln C_{{\text{e}}}$ will yield a straight line of slope equal to $\frac{RT}{b}$ and intercept equal to $\frac{RT}{b}{\ln}K_{{\text{T}}}$ from which the constants can be determined if the adsorption follows the Temkin model.

**Fig. 5:**
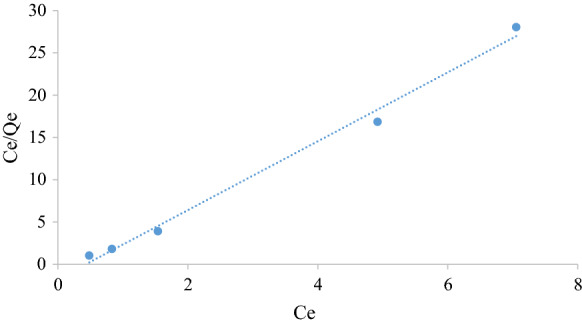
Langmuir batch isotherm

**Fig. 6:**
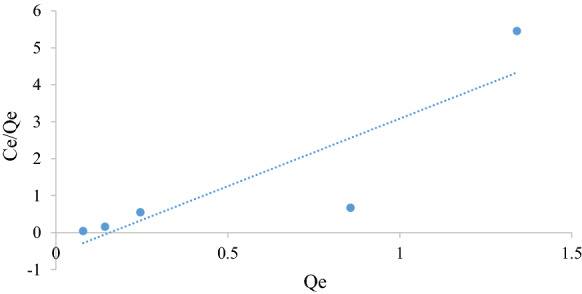
Langmuir column isotherm

**Fig. 7:**
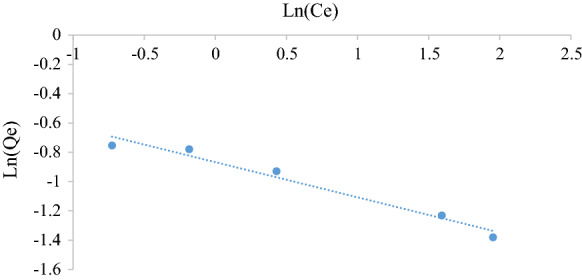
Freundlich batch isotherm

**Fig. 8:**
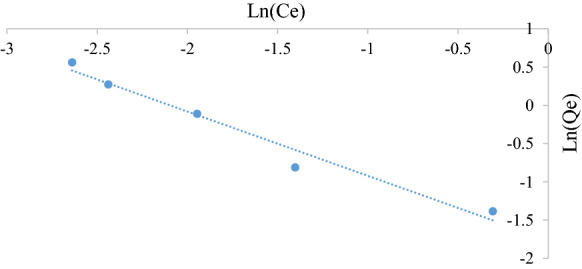
Freundlich column isotherm

**Fig. 9:**
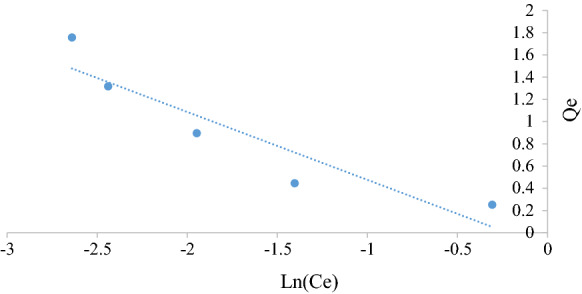
Tempkin batch isotherm

**Fig. 10:**
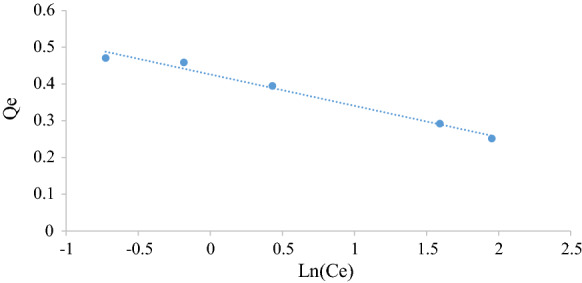
Tempkin column isotherm

Figures 5, 6, 7, 8, 9 and 10 show the respective Langmuir, Freundlich and Temkin isotherms with a variation in adsorbent dosages (1.5–20 g) at neutral pH and 22 °C when using batch and column configurations. Upon closer inspection of the *R*^2^ value, the Freundlich model most accurately represents the adsorption process of both the batch and column configuration. However, the Langmuir model also has a good *R*^2^ value. However, due to a negative slope resulting in a negative value of the Langmuir constant (*k*_L_) being physically impossibly, this model is not an accurate representation and the adsorption does not follow this isotherm (Kiurski et al. 2011). The constant, n, from the Freundlich model is greater than 1, which indicates favourable adsorption. The negative value of the Temkin constant, which signifies the heat of adsorption, also indicates that the process is endothermic. The constant parameters were all determined by the respective isotherm plots and are summarized in Table 3.

**Table 3:** Langmuir, Freundlich and Tempkin adsorption isotherm constants

	Langmuir			Freundlich			Tempkin		
	*k*_L_	*q*_m_ (mg g^−1^)	*R*^2^	*k*_F_ (mg/g)	*n*	*R*^2^	*k*_T_	*b* (kJ/mol)	*R*^2^
Batch	− 2.32	0.25	0.9923	0.42	4.16	0.969	1	− 29.00	0.982
Column	− 0.69	0.69	0.7608	0.17	1.19	0.9694	1	− 4.10	0.8558

### Thermodynamics

The thermodynamics of the adsorption process can be analysed in order to determine the mechanism, such as whether the process is physical or chemical, spontaneous or non-spontaneous and exothermic or endothermic. Adsorption studies need to be conducted at different temperatures in order to determine parameters such as the activation energy (*E*_a_), change in standard free energy (∆*G*°), enthalpy (∆*H*°) and entropy (∆*S*°). These parameters can be estimated using Eqs. (8), (9) and (10).

8 $$K_{{\text{C}}} = \frac{{q_{{\text{e}}} }}{{C_{{\text{e}}} }}$$

9 $${\ln}K_{{\text{c}}} = \frac{{\Delta S^{^\circ } }}{R} - \frac{{\Delta H^{^\circ } }}{RT}$$

10 $$\Delta G^{^\circ } = - RT{\ln}K_{{\text{c}}}$$

where *R* is the universal gas constant (8.314 J mol^−1^ K^−1^), T is the absolute temperature (K), ∆*H*° is the standard enthalpy (kJ mol^−1^), ∆*S*° is the standard entropy (kJ mol^−1^), and ∆*G*° is the standard free energy change (kJ mol^−1^). Values for ∆*H*° and ∆*S*° can be calculated from the slope and intercept of a linear plot of $\ln K_{{\text{c}}}$ and $\frac{1}{T}$ (Banerjee et al. 2013). Estimated values for these parameters are tabulated and shown in Table 4.

**Table 4:** Thermodynamic parameters for the adsorption of MO on fly ash at 25 °C

Temp (°C)	∆*G*° (kJ mol^−1^)	∆*H*° (kJ mol^−1^)	∆*S*° (kJ mol^−1^)
20	− 9.36	13.36	77.75
25	− 9.84		
30	− 10.29		
35	− 10.68		
40	− 10.88		

The negative value of ∆*G*° for all temperatures indicates that the adsorption process is spontaneous. The positive ∆*H*° value as well as the increase in ∆*G*° values with an increase in temperature further shows the process is endothermic, as adsorption is favoured at higher temperatures. This is most likely due to the fact that the mobility of the adsorbate increases with an increase in temperature and the affinity of the adsorbate increases at higher temperatures (Saha and Chowdhury 2011). A positive ∆*S*° suggests that the process follows a dissociative mechanism, i.e. that there is an affinity of the adsorbent for the adsorbate, as well as an increases in the degree of freedom accompanied by an increase in randomness at the solid–liquid interface (Saha and Chowdhury 2011). The magnitude of ∆*G*° and ∆*H*° indicates that the process is physical as ∆*G*° is in the range of 0–20 kJ mol^−1^ (Salam et al. 2012) and ∆*H*° in the range of 2.1–20.9 kJ mol^−1^ (Liu and Liu 2008). Similar findings were obtained by Jalil et al. (2010) and Yang et al. (2016) for other low-cost adsorbents using MO.

**Fig. 11:**
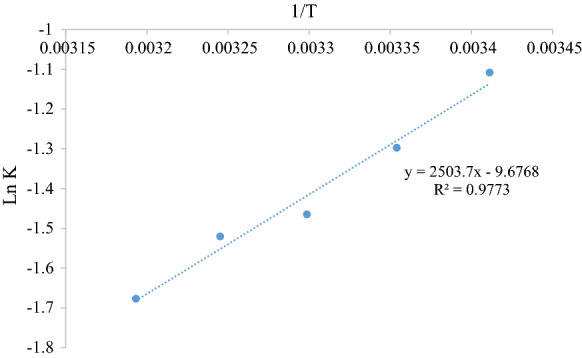
Activation energy plot for the adsorption on MO on fly ash

The pseudo-second-order rate constants from the Lagergren model (*k*_2_) evaluated at different temperatures using a batch configuration were used to determine the activation energy of the adsorption process by applying the Arrhenius equation given by Eq. (11). A plot of ln *k* versus $\frac{1}{T}$ is shown in Fig. 11.

11 $$\ln k_{2} = \ln A - \frac{{E_{{\text{a}}} }}{RT}$$

where *E*_a_ is the activation energy (kJ mol^−1^) and A is the Arrhenius factor (g mol^−1^ s^−1^).

The activation energy was calculated to be − 20.82 kJ mol^−1^. This value is fairly small, which is an indication that the adsorption may not be very sensitive to the experimental temperature range. The low value (*E*_a_ < 42 kJ mol^−1^) indicates that the adsorption is physical (Umpuch and Sakaew 2013). The negative activation energy implies that the rate of adsorption decreases with an increase in temperature which leads to a reduction in the probability of the colliding molecules being captured by the adsorbent. This negative value also indicates that energy barriers are absent in this process (Kobiraj et al. 2012).

### Column, batch and heap adsorption comparison

**Fig. 12:**
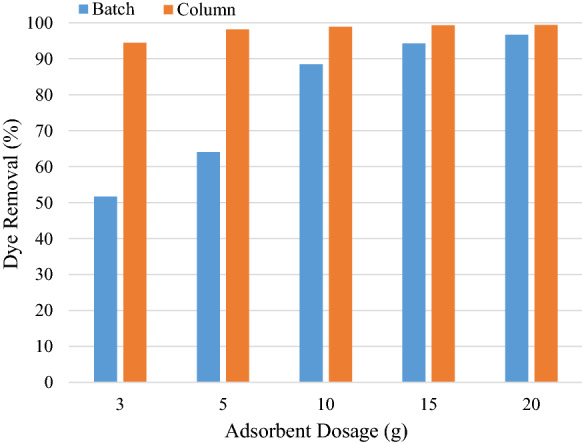
Dye removal comparison between batch and column configurations

Figure 12 shows the dye removal comparison between batch and column operation using the same solution volume, adsorbent dosage as well as pH.

It can be seen that the column configuration has a much larger dye removal percentage. This high removal efficiency on the column operation was largely dependent on the bed height, with the largest adsorbent dosage having the greatest removal efficiency due to longer residence times and increased number of binding sites (Olgun et al. 2013). Removal percentages became stagnant beyond a certain mass of adsorbent as binding sites were restricted due to tight column packing (Chen et al. 2011).

Heap adsorption showed an overall dye removal of 99.25% after 3 washing cycles compared to a column with a maximum removal of 99.95% and batch with 96.68%. Both the column and heap adsorption are comparable in performance. Each configuration has their own drawbacks, as heap adsorption requires a large adsorbent mass and area, whereas column operations have long residence times and batch operations are energy intensive.

### Adsorption capacity

The maximum adsorption capacity of the column (8.9 mg g^−1^) is higher than that of the batch process (3.9 mg g^−1^) under the same operating conditions. This may be due to the surface of coal fly ash, which favours solid-state diffusion relative to the batch configuration. Similar results of higher column adsorption capacities were reported by (Gupta et al. 2001). Heap adsorption has an intermediate adsorption capacity of 4.0 mg g^−1^. Fairly low adsorption capacities are indicative of an anionic dye since the presence of a negatively charged carboxyl group, which is an important functional group present in industrial effluents, inhibits the adsorption of anionic dyes (Gong et al. 2005). Table 5 shows a comparison of adsorption capacities found by similar studies using an anionic dye under batch configuration.

**Table 5:** Adsorption capacity comparison between varying adsorbents and anionic dyes

Adsorbent	Anionic dye	Adsorption capacity (mg g^−1^)	References
Fly ash	Methyl orange	3.9	Present study
Fly ash	Reactive blue 171	3.75	Banerjee et al. (2014)
Carbon nanotubes	Methyl orange	44.16	Zhao et al. (2013)
Peanut Hull	Sunset yellow	13.99	Wang and Zhu (2005)
Coal fly ash-NaOH	Acid red 1	12.66	Hsu (2008)
Fly ash	Acid red 91	1.75	Ramakrishna and Viraraghavan (1997)
Chitosan beads	Remazol blue	201.6	Pereira et al. (2017)
Cross-linked chitosan	Methyl orange	89.29	Huang et al. (2017)
Eggshells	Congo red	49.5	Abdel-Khalek et al. (2017)
ZnO nanoparticles	Congo red	71.43	Kataria and Garg (2017)

Adsorption capacities differ drastically due to different sample pre-treatment, pH, contact times and temperatures. One major difference is the particle size of the coal fly ash. Since a small surface area of 1.42 m^2^ g^−1^ was found, there were less active sites present for adsorption resulting in the low adsorption capacity. However, in comparison, the adsorption capacity of coal fly ash is in a fairly intermediate range and can be considered a viable alternative for dye removal.

### Economic evaluation

Despite column operation being the most efficient dye removal technique, each method has various economic constraints. On the assumption that 100 Ml of water needs to be treated daily, with dye concentration of 500 ppm to be reduced to 1 ppm, Table 6 summarizes the costs and mass required for each operation. Coal fly ash was assumed to have no cost associated with it, and only logistical costs such as transportation need to be considered and were estimated based on the mass of adsorbent required (Freight Rate Calculator 2017). The mass of coal fly ash required is determine from the respective adsorption capacities.

**Table 6:** Economic evaluation for various operating conditions

	Batch	Column	Heap leaching
Maximum adsorption capacity (mg/g)	3.9	8.9	4.0
Mass of fly ash required (Tonnes/Day)	128	56	125
Cost of treatment (R/day)	2500	1094	2441

Column operation is the cheapest due to the smaller adsorbent dosage required. Various government grants in South Africa are awarded to wastewater treatment plants such as the Regional Bulk Infrastructure Grant which allocates an excess of 5 billion rand to various wastewater treatment companies (van Zyl et al. 2016). Other revenue sourced from industries is the premium paid to treat or dispose wastewater effluents. While the use of coal fly ash seems feasible, further studies at larger scale, such as a pilot plant, needs to be conducted.

## Conclusions

The study shows that coal fly ash, which is often applied for cationic dye removal, is also a suitable adsorbent for the removal of the anionic dye, methyl orange. The adsorption process follows pseudo-second-order kinetics and the Freundlich isotherm most accurately represents the adsorption for both batch and column configurations. Upon analysis of the thermodynamic parameters, negative values of the Gibbs free energy change indicate the process is spontaneous. Positive enthalpy and entropy changes indicate that the adsorption is endothermic, random and a physical process. The adsorption process has an activation energy of − 20.82 kJ mol^−1^, suggesting that energy barriers are very low or absent. Of the three configurations, dye adsorption is the highest using column operation, followed by heap adsorption and lastly a batch operation.
